# Healthy human brains have a daily heatwave

**DOI:** 10.1080/23328940.2022.2150040

**Published:** 2023-06-15

**Authors:** Nina M Rzechorzek, John S O’Neill

**Affiliations:** 1 MRC Laboratory of Molecular Biology, Cambridge, CB2 0QH, UK

In summer 2022, parts of the United Kingdom hit record temperatures above 40°C. As it turns out, parts of the human brain are reaching these heights on a daily basis [1], and this could be vitally important for its optimal function.

Almost all biological processes are temperature-sensitive and neuronal activity is no exception [2]. Clearly, there is an optimal range of temperatures over which the brain operates best. A voracious energy consumer, the human brain receives 20% of cardiac output at rest and generates a sixth of our metabolic heat, whilst representing just 2% of body mass. If not dealt with, this heat is expected to result in biochemical failure, and brain damage. Logically, neuroprotective cooling aims to reduce the metabolic demand of the brain when supply is compromised. Indeed, hypothermic preconditioning can protect human neurons *in vitro* from stress [3] and, for specific patient groups, there is robust evidence supporting the use of therapeutic hypothermia [4].

Given its importance to brain health, decades of assumption have entrenched the idea that brain temperature (*T*_Br_) is static, homogenous, and matches other parts of the body’s core. Yet, these notions are in conflict with each other—human gut temperature irrefutably varies in a daily manner, even in temperature-controlled environments [5]. Daily variations in *T*_Br_ are demonstrable in rodents and non-human primates; in the latter, *T*_Br_ is consistently higher than carotid artery, aortic arch, and abdominal cavity temperature, and exhibits its own spatial gradient [1]. Some clinical studies report that *T*_Br_ increases after brain injury and some patients undergo interventions to achieve a ‘normal’ *T*_Br_. The rationale for this approach is problematic because (astonishingly) we don’t know what normal human *T*_Br_ is, or how much it varies. *T*_Br_ can only be measured directly in human brains that are already injured, so interpretation of these data is hampered by the lack of a healthy reference dataset. To address this gap, we undertook a retrospective analysis of *T*_Br_ measured directly in patients with traumatic brain injury, alongside a prospective study of *T*_Br_ measured non-invasively in healthy adults using magnetic resonance spectroscopy (MRS) [1].

First, we screened the Europe-wide CENTER-TBI database for patients who had undergone direct *T*_Br_ monitoring without receiving temperature-based interventions. In our cohort of 114 patients, *T*_Br_ exceeded temperature measured at other body locations and ranged from 32.6 to 42.3°C. Only 25% of patients displayed a daily rhythm *T*_Br_ and their temperature maxima and minima were poorly aligned with external time. We noted that *T*_Br_ range decreased with age, suggesting a reduced variability in older patients. Next, we recruited 40 healthy adults aged 20-40 years for brain imaging. Participants were scanned in the morning, afternoon, and late evening of a single day, immediately after one week of actigraphy. This allowed us to control for differences in chronotype—how an individual’s body clock aligns with the day-night cycle—which could have masked a true daily variation in *T*_Br_ across the group. We used a validated MRS technique to measure *T*_Br_ in several locations within the cerebrum and deeper areas including the thalamus and hypothalamus—a key brain structure involved in biological timing and thermoregulation. In healthy adults, mean *T*_Br_ exceeded oral temperature and varied spatially by 2.4°C with highest temperatures in the thalamus, the most central brain region measured. Across the cohort and all time points, *T*_Br_ ranged from 36.1 to 40.9°C, whilst oral temperature varied less (34.6 to 37.0°C). Notably, *T*_Br_ increased with age, especially in deep brain regions with a 0.6°C increase between ages 20 and 40. *T*_Br_ varied by time of day, being lowest at night in both sexes, but on average it was 0.4°C higher in post-ovulation females relative to pre-ovulation females and males, and this difference was exaggerated 2-fold in deep brain regions. The time of day variation was also greatest in deep brain regions with nearly a 1°C drop by midnight.

We used our MRS data to model *T*_Br_ over a complete day-night cycle to produce HEATWAVE—the first 4D map of normal human *T*_Br_. HEATWAVE has yielded results that are physiologically intuitive, yet neurologically surprising. Human *T*_Br_ is clearly higher and varies more than previously assumed. Sex differences appear to be driven by menstrual cycle phase, whilst time of day variation reflects established daily rhythms in temperature measured in other parts of the body. Since cerebral blood flow is key to brain heat transfer, a nightly fall in *T*_Br_ temporally aligns with increased cerebral blood flow during sleep [1] and temperature gradients across the brain agree with predictions from cerebrovascular anatomy and thermodynamics. Age-related changes in cerebrovascular function might underlie progressive impairment of brain cooling—but this remains to be formally tested. Conceptually, an age-related *T*_Br_ increase should be partially offset by advancing brain atrophy and the loss of heat-generating tissue, leading to nonlinearity in later years. An important question emerging from our data is whether the daily *T*_Br_ range would decrease in the aged brain, mirroring a reduced amplitude in temperature rhythms observed in other parts of the body [1]. In this regard, the wider physiological state of an individual (in particular hydration and acclimatization) would be expected to influence thermoregulatory capacity and thus *T*_Br_ extremes over a 24-hour period. The marked spatial distribution of *T*_Br_ presents the conceptual challenge of how neural circuits spanning such a temperature gradient could deal with, or indeed exploit, it. More perplexing are the larger temperature gradients that might exist within the longest cells in the body. The selective vulnerability of certain motor neuronal subtypes in some of the most devastating neurological disorders may be compounded or partially explained by spatiotemporal temperature variation.

Spatiotemporal variation in *T*_Br_ is likely to have a complex, and perhaps reciprocal relationship with sleep. The mechanistic basis of sleep and its purpose are uncertain, but our data are consistent with a thermal hypothesis for sleep function and evolution, wherein *T*_Br_ decline is permissive for entry into non-rapid eye movement sleep, whilst rapid eye movement sleep serves to keep *T*_Br_ above a vital threshold [6]. Sleep disturbance in hot weather might thus represent a failure of *T*_Br_ to fall at the required rate, or reach the required minima for some sleep stages. Intriguingly, HEATWAVE predicts a greater daily excursion of temperature in deep brain regions in males; future studies should extend measurement into other parts of the brainstem that play a role in sleep. *T*_Br_ dynamics illuminated by HEATWAVE ultimately demand a reappraisal of how daily neuronal activity is regulated and, in effect, how the brain works. In proposing such a paradigm shift, parallels can be drawn with the derailment of Galenic doctrine which asserted that humans had an intracranial *rete mirabile*. For much of the sixteenth and seventeenth centuries this intricate brain-cooling vasculature was falsely depicted as a feature of human anatomy based on its demonstration in other species [7]. This costly mistake, perpetuated by Galen’s acolytes, exemplifies the dangers of reliance on established authority over direct observation. Challenging embedded beliefs, our findings can stimulate further research into the mechanistic underpinnings of *T*_Br_ in health and disease.

Having established a baseline for normal human *T*_Br_ and its spatiotemporal variation—what does this mean for patients? Evidently, our retrospective dataset contained injured brains that were above, below, or within the normal range of *T*_Br_, but not necessarily all of the time. Looking again at these data we tested which features of *T*_Br_ were related to outcome. Ageing by 10 years increased the odds of death 11-fold and a warmer mean *T*_Br_ was associated with survival, but neither temperature maxima, minima, nor range predicted mortality. Most strikingly, however, lack of a daily *T*_Br_ rhythm increased the odds of death in intensive care 21-fold [1]. In real terms, of 98 patients in our outcome analysis (Fig.1), 21 died and only one of these had a daily rhythm in *T*_Br_. By contrast, of the 77 who survived, 24 had a daily rhythm in *T*_Br_. Thus, it seems that *T*_Br_ variation, rather than absolute *T*_Br_, better distinguishes brain function and dysfunction. The ‘take home’ is not that the lack of a daily *T*_Br_ rhythm reliably predicts death, but that the presence of this rhythm alone is a positive sign that should be factored into decision making. Whilst larger prospective studies are needed to validate these results, our findings extend the prognostic power of *T*_Br_ and raise questions about how patient temperature is currently managed and interpreted—not least that the *T*_Br_ recorded from a patient depends on factors unrelated to their brain injury. HEATWAVE thus provides a valuable reference dataset for future studies in different age groups and patient cohorts. For example, *T*_Br_ variability might predict and influence the manifestation of chronic brain disorders. Indeed, we can readily envisage clinical trials to assess the utility of *T*_Br_ rhythm disruption as an early biomarker for neurodegenerative disease. Achieving this at scale will require the development of cost-effective, practical, and non-invasive technologies to capture longitudinal variations in temperature across the brain.

Abnormal body temperature rhythms feature early in neurodegenerative disorders and are considered a manifestation of disrupted sleep and circadian cycles. If *T*_Br_ rhythms are similarly affected, it will be critical to establish how normal *T*_Br_ variation interacts with the daily neural molecular clockwork before inferring a role for *T*_Br_ disruption in disease. To this end, HEATWAVE is transforming how we explore neural circadian oscillations in the lab, and how key molecular components of these biological clocks operate within the rhythmic biophysical environment of the cell. Whilst these new data raise more questions than they answer, the human brain can no longer be viewed as an isothermal machine.

## Figures and Tables

**Fig. 1:**
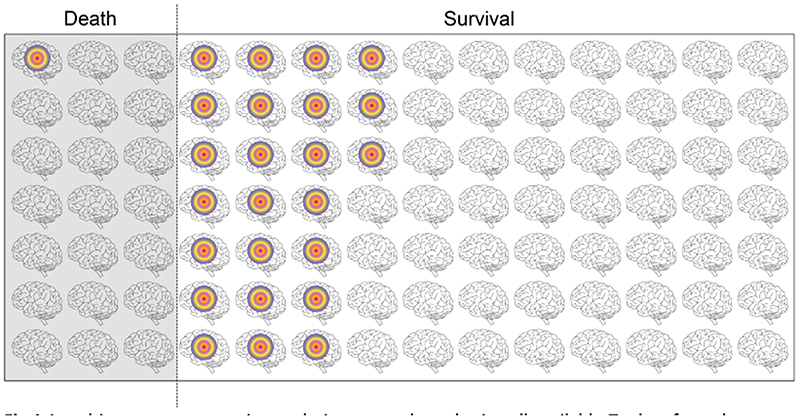
A multicentre, retrospective analysis was conducted using all available *T*_Br_ data from the CENTER-TBI High Resolution ICU Sub-Study [1]. These data were collected at a minimum of 1-min intervals from a single location in frontal white matter (around 18 mm below the dura), in patients with moderate to severe brain trauma. Data collected in the context of Targeted Temperature Management were excluded. Rhythmicity analyses were performed on datasets where at least 36 h of continuous data were obtained, using strict criteria and a multiplexed algorithm approach [1]. A generalized linear mixed model was applied to determine whether there was any relationship between *T*_Br_ features and patient outcome. Patients with a daily rhythm in brain temperature (coloured circles) had a greater chance of survival in intensive care (*P*=0.016; *n*=98).
